# Nanomaterial based photoacoustic interface for non-genetic neuromodulation

**DOI:** 10.1088/2399-1984/ae44dc

**Published:** 2026-03-04

**Authors:** Deming Li, Guo Chen, Yueming Li, Nan Zheng, Zhiyi Du, Chen Yang

**Affiliations:** 1 Department of Electrical and Computer Engineering, Boston University, Boston, MA, United States of America; 2 Division of Materials Science and Engineering, Boston University, Boston, MA, United States of America; 3 Department of Chemistry, Boston University, Boston, MA, United States of America

**Keywords:** nanomaterial, photoacoustic, neural stimulation, non-genetic neuromodulation, brain, retina

## Abstract

Photoacoustic (PA) stimulation is an emerging technology aiming to modulate neuronal activity safely, precisely, and efficiently without genetic modification. Owing to their strong light absorption, easy to process in the composite, nanomaterials have been utilized as PA interfaces developed for high spatial and temporal resolution PA stimulation without thermal damage *in vitro* and *in vivo*. This topical review introduces the theory of PA generation and summarizes the nanomaterials used in PA neural modulation. We discuss how these PA interfaces are applied in *in vitro* and *in vivo* studies, and highlight their unique capabilities and potential clinical applications. We further review mechanistic studies that provide insights into how neurons respond to mechanical stimuli. Overall, nanomaterial-based PA neural interfaces have shown exciting potential in non-genetic modulation of the brain and retina, serving as a unique tool for neuroscience and opening new opportunities for treating neurological disorders.

## 1. Introduction

High precision neuromodulation is an essential tool in neuroscience to understand how neurons and neural systems work. It also offers an alternative potential treatment of neurological disorders in patients otherwise do not respond to drugs [1, 2]. Electrical stimulation, such as electrode-based deep brain stimulation, has been used for treating neurological diseases clinically [3, 4]. However, the spread of the electric current limits its spatial resolution at the millimeter level, and the electronic components raise concerns about long-term stability and functional magnetic resonance imaging (fMRI) compatibility. Transcranial stimulation methods including transcranial magnetic stimulation, transcranial direct current stimulation, and transcranial focused ultrasound have also been used in clinical applications and tested in clinical trials respectively. They are noninvasive but offer the spatial resolution only at the millimeter level [5, 6]. Optogenetics has shown successful population neural stimulation and provided cell specificity [7, 8]. Yet, the requirement for viral infection hinders its further applications in primates and humans. Photothermal (PT) stimulation has a high spatial and temporal resolution, but the rapid temperature change may cause tissue damage [9]. New non-genetic neural stimulation with high precision, biocompatibility, and translational potential is still in critical need.

Photoacoustic (PA) neural stimulation is an emerging non-genetic method with high precision up to the single-cell level. PA interfaces with light absorbing materials convert nanosecond laser pulses to transient heating and result in thermal expansion, generating pulsed acoustic waves at ultrasonic frequencies [10, 11]. It has been found that wild-type neurons in rodent brain and retina respond to PA signals. *In vitro* and *in vivo* PA neural stimulation has been achieved using PA signals with frequencies ranging from a few hundred kilohertz to tens of megahertz and pressure ranging from a few hundred kilo-pascals to about a few megapascals. Significantly, since the generated acoustic waves have wavelengths less than the emitter dimension, the PA emitter acts like a point source, producing acoustic waves propagating omnidirectionally. This results in a tightly confined ultrasound field and leads to high precision neural stimulation at single-cell and sub-cellular resolution. In addition, studies using continuous wave (CW) laser and nanosecond pulsed laser indicated that PA stimulation of neurons in culture achieved by the pulsed laser requires about 30–40 times less energy than PT stimulation achieved by the CW laser [12, 13]. While the cellular mechanism of the difference is still under investigation, this finding indicated that PA stimulation can be potentially safer than PT stimulation. It is also worth highlighting that various platforms have been designed to utilize PA effect with biocompatibility, flexibility, miniaturization, and fMRI compatibility, which offer additional engineering advantages when applying them to modulate neural systems.

Specifically, in the design of these PA interfaces, nanomaterials play an important role. Nanomaterial as a highly efficient optoacoustic converter has been previously used for PA generation, including all-optical ultrasound imaging [14, 15], tissue cavitation [16, 17], and precision surgical guidance of lumpectomy [18]. From the perspective of material, nanomaterials such as gold nanorods [19, 20], carbon nanotubes (CNTs) [16], Si nanowires [21, 22], and polymer nanoparticles [23], have a strong optical absorption, which can act as localized PA sources to generate strong ultrasound pressure. These materials convert photons into acoustic waves efficiently and can be applied to specific regions, improving the depth, resolution, and safety of PA neuromodulation. From the perspective of engineering, nanomaterials are easy to process in the composite and therefore offer the flexibility to design advanced coating structures, such as multilayers or mixture materials, to further enhance PA conversion efficiency. Additionally, incorporating biocompatible polymers or biomaterials into the mixture or composite can enhance biocompatibility and achieve seamless integration with biological tissue. Our team developed various PA devices based on nanomaterials for neuromodulation, including fiber-based PA emitters, film-based PA devices, and PA nanoparticles, all of which have been successfully applied both *in vitro* and *in vivo*.

In this topic review, the fundamental theory of PA generation will be introduced at first. Then, the different nanomaterials used in PA interfaces for different applications will be summarized and compared. The main part of this review is the PA neural stimulation achieved *in vitro* and *in vivo* studies. The unique capabilities and functions of the different interfaces will be highlighted. Current understanding of the PA cellular mechanism will be presented. Last but not least, the remaining challenges will be discussed, and an outlook will be provided for the future development of PA neural stimulation technology.

## 2. Overview of PA effect

The PA effect arises when a pulsed laser is absorbed by the absorbers and converted into heat and mechanical energy in the form of a broadband ultrasonic wave. The PA effect is the foundation of PA neuromodulation.

### 2.1. The theory of PA generation

Following the theory established by Wang [10, 24, 25], for absorbers to efficiently generate a PA wave, thermal confinement and stress confinement conditions need to be met. When the duration of the laser pulse is shorter than the thermal and stress dissipation time, the heat conduction or stress propagation is negligible during the laser pulse, ensuring efficient generation of PA. The thermal dissipation time ${\tau _{{\mathrm{th}}}}{ }$ is given as:

\begin{equation*} {\tau _{{\mathrm{th}}}} = \frac{{d_{\mathrm{c}}^2}}{{{\alpha _{{\mathrm{th}}}}}} \end{equation*}

where the ${\alpha _{{\mathrm{th}}}}$ is thermal diffusivity and *d*_c_ is the characteristic dimension of the heated region. The stress dissipation time ${\tau _{\mathrm{s}}}{ }$ is defined as:

\begin{equation*} {\tau _{\mathrm{s}}} = \frac{{{d_{\mathrm{c}}}}}{{{v_{\mathrm{s}}}}} \end{equation*}

where the ${v_{\mathrm{s}}}$ is the speed of sound in the medium. For example, in a candle soot fiber-based optoacoustic emitter (CSFOE) with the thickness of candle soot (CS), as the absorber, of 10 *μ*m, the stress dissipation time is estimated to be 9 ns and the thermal dissipation time is estimated to be 909 ns. Since the two dissipation times are at the nanosecond level, a nanosecond pulse laser is commonly used to generate a PA signal.

When a nanosecond pulsed optical excitation is delivered to the absorber, the absorbed optical energy is converted into local heating, which induces a transient temperature rise and mechanical expansion as well as compression. Upon excitation, the change of the fractional volume can be expressed as:

\begin{equation*} \frac{{{\mathrm{d}}V}}{V} = - \kappa p + \beta T \end{equation*}

where *β* is the thermal coefficient of volume expansion (K^−1^), $p$ and *T* denote the changes in pressure (Pa) and temperature (*K*), respectively. ${ }\kappa = { }\frac{{{C_{\mathrm{p}}}}}{{{C_{\mathrm{v}}}\rho v_{\mathrm{s}}^2}}$ is the isothermal compressibility (Pa^−1^), with *v*_s_ being the speed of sound, *C*_p_ and *C*_v_ being the specific heat capacity at constant pressure and volume, respectively.

In both the thermal and stress confinements, the fractional volume expansion is negligible, and the local pressure rise *p*_0_ immediately after the laser pulse can be derived from (3) as

\begin{equation*} {p_0} = \frac{{\beta T}}{\kappa } = \Gamma {\eta _{{\mathrm{th}}}}{\mu _{\mathrm{a}}}F\end{equation*}

where ${\eta _{{\mathrm{th}}}}$ is the percentage of energy that is converted into heat, *μ*_a_ is the optical absorption coefficient, and *F* is the optical fluence (J m^−2^). $\Gamma = \frac{{\beta v_{\mathrm{s}}^2}}{{{C_{\mathrm{p}}}}}$ is the Grueneisen parameter, which depends on material properties.

The generation and propagation of the PA pressure wave in an inviscid medium is governed by the general PA wave equation:

\begin{equation*} {\nabla ^2}p\left( {\vec r,t} \right) - \frac{1}{{v_{\mathrm{s}}^2}}\frac{{{\partial ^2}p\left( {\vec r,t} \right)}}{{\partial {t^2}}} = - \frac{\beta }{{\kappa v_{\mathrm{s}}^2}}\frac{{{\partial ^2}T\left( {\vec r,t} \right)}}{{\partial {t^2}}} \end{equation*}

where $p\left( {\vec r,t} \right)$ denotes the acoustic pressure at location $\vec r$ and time *t*, and *T* denotes the temperature. The left-hand side of this equation describes the PA wave propagation, while the right-hand side describes the source.

In thermal confinement, the thermal function can be described by the converted thermal energy:

\begin{equation*} H\left( {\vec r,t} \right) = \rho {C_{\mathrm{v}}}\frac{{\partial T\left( {\vec r,t} \right)}}{{\partial t}}.\end{equation*}

Then we can simplify (5) as:

\begin{equation*} {\nabla ^2}p - \frac{1}{{v_{\mathrm{s}}^2}}\frac{{{\partial ^2}p}}{{\partial {t^2}}} = - \frac{\beta }{{{C_{\mathrm{p}}}}}\frac{{\partial H}}{{\partial t}}.\end{equation*}

The equation means that the wave propagation is only related to the first time derivative of heating. Therefore, time-invariant heating does not produce a pressure wave; only time-variant heating does.

Under the condition of a finite pulsed excitation, thermal confinement, monopole radiation, and zero ultrasonic attenuation loss, we can apply the Green’s function to the general equation to solve the forward solution in the time domain and frequency domain as:

\begin{equation*} {p_{\delta D}}\left( {\vec r,t} \right) = \frac{1}{{4\pi v_{\mathrm{s}}^2}}\frac{{{p_0}}}{{\left| {\vec r} \right|}}\frac{{{\mathrm{d}}I\left( {t - \frac{{\left| {\vec r} \right|}}{{{v_{\mathrm{s}}}}}} \right)}}{{{\mathrm{d}}t}}\end{equation*}

\begin{equation*} {p_{\delta D}}\left( {\vec r,\omega } \right) = - \frac{{{\mathrm{i}}\omega \beta {\mu _{\mathrm{a}}}\bar I{\eta _{{\mathrm{th}}}}}}{{4\pi {C_{\mathrm{p}}}}}\frac{{{{\mathrm{e}}^{{\mathrm{S}}k\left| {\vec r} \right|}}}}{{\left| {\vec r} \right|}}.\end{equation*}

Equations (8) and (9) can help us have a better understanding of the contributing factors to the generated PA pressure. In the time domain, the monopole PA amplitude is proportional to the initial pressure ${p_0}$ at the origin and the first derivative of the excitation pulse temporal profile *I(t)*. This implies that to get a strong PA pressure ${p_{\delta D}}\left( {\vec r,t} \right)$, apart from using a high incident laser power, we can choose nanomaterials with a high optical absorption coefficient or a large Grüneisen parameter to increase ${p_0}.$ In the frequency domain, the PA amplitude is proportional to the modulation frequency $\omega $ at the origin. Here, the modulation frequency is related to the laser pulse width, which indicates that using an incident laser with a shorter pulse width leads to a stronger PA pressure.

### 2.2. Measurement of PA signal generated by fabricated emitters

**Figure 1.**
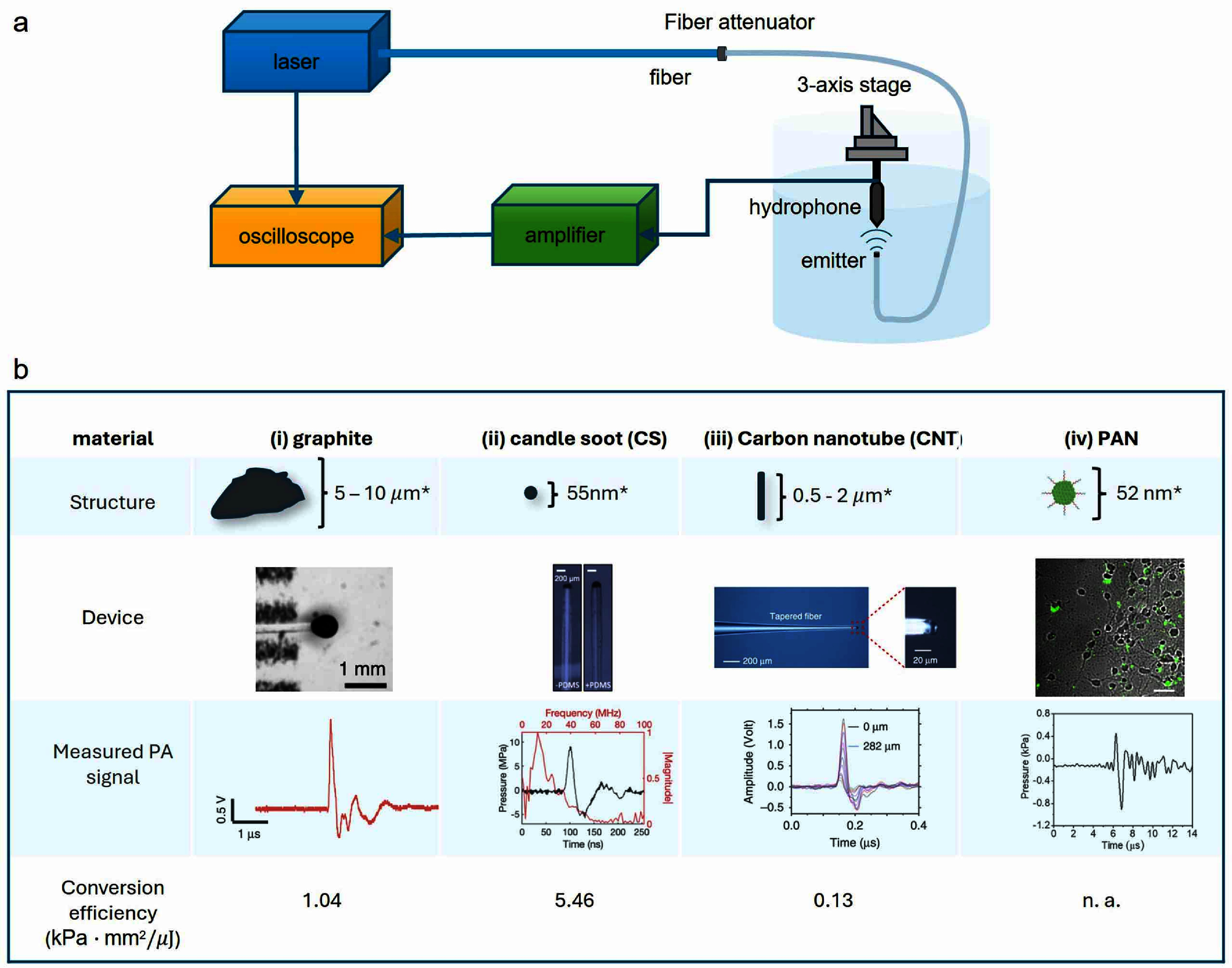
(a) Schematic of a typical PA measurement and (b) nanomaterials used in PA stimulation. i) Schematic of particle shape and diameter of graphite, photo of FOC (scale bar: 1 mm), PA waveform, and conversion efficiency. The generated pressure has a central frequency of 1.4 MHz, and the input pulse energy is 14.5 ${{\mu J}}$. ii) Schematic particle shape and diameter of candle soot, photo of CSFOE before and after PDMS coating (scale bar: 200 ${\text{ }}{{\mu m}}$), PA waveform with frequency spectrum and conversion efficiency. The generated pressure has a central frequency of 12.8 MHz, and the input pulse energy is 56 ${{\mu J}},{\text{ }}{{measured}}{\text{ }}{{at}}{\text{ }}70$ ${{\mu m}}$. iii) Schematic particle shape and diameter of carbon nanotube, photo of TFOE (scale bar: left: 200${\text{ }}{{\mu m}},{\text{ }}{{right}}:{\text{ }}20{\text{ }}{{\mu m}}$), PA waveform, and conversion efficiency. The generated pressure has a central frequency of 6.6 MHz (at 0 ${{\mu m}}$), and the input pulse energy is 6.7 *μ*J. The curves from top to bottom were measured at the distances of 0, 2, 6, 10, 17, 29, 49, 82, 145, 185, and 282 *μ*m, respectively. iv) Schematic shape and diameter of a polymer nanotransducer, TA images of PANs binding to neurons after 15 min culture (Green, TA channel; gray, transmission channel; scale bar: 50 ${\text{ }}{{\mu m}}$), PA waveform, and conversion efficiency. The generated pressure is measured in PAN solution (1 mg ml^−1^), and the laser power density is 21 mJ cm^−2^. All of the PA pressures were measured by a 40 ${{\mu m}}{\text{ }}$ needle hydrophone (Precision Acoustics, UK), generated from a *Q*-switched diode-pumped solid-state laser (PQSY, RPMC USA) centered at 1030 nm with a pulse width of 3 ns. *The size of graphite and CNT is from the vendor, the size of candle soot is from [26], the size of the polymer nanotransducer. Reproduced from [27], with permission from Springer Nature. Reproduced from [28]. CC BY 4.0. Reproduced from [29], with permission from Springer Nature. Reprinted from [30], Copyright (2021), with permission from Elsevier.

The setup of a typical PA measurement is shown in figure 1(a). To generate the optoacoustic signal, a nanosecond pulse laser driven by a waveform generator was used as the excitation laser source. The laser was first connected to a 200 *μ*m optical fiber and then connected to a PA emitter. Fiber optic attenuator sets were used to adjust the pulse energy. Upon pulsed laser excitation, the absorbed optical energy was rapidly converted into heat by the optoacoustic emitters, resulting in transient expansion and the generation of pulsed and broadband ultrasonic waves. The generated acoustic signal propagated through the surrounding medium and was measured through a homebuilt system including a hydrophone or an ultrasound transducer fixed on a 3-axis stage, an amplifier, and an oscilloscope. The time-domain data represented the pressure values, which were calculated based on the calibration factor provided by the hydrophone/transducer manufacturer. The frequency-domain data were obtained by performing a fast Fourier transform calculation using MATLAB R2023b.

Noise in the PA measurements arises from optical, electronic, and environmental sources. Optical noise mainly comes from laser pulse-to-pulse energy fluctuations, which can be minimized through ensemble averaging over multiple laser pulses. Electronic noise originates from preamplifier and oscilloscope noise and impedance mismatch, and can be decreased by proper termination and connecting a high pass filter or amplifier. Environmental and mechanical noise, including vibrations, water motion and air bubbles, can be suppressed by using degassed water as the measurement medium and vibration-isolated setups. In addition, post-processing techniques such as bandpass filtering and signal averaging will further improve the signal-to-noise ratio. In our measurements, the typical signal-to-noise ratio is 48–54 dB after averaging, indicating that the noise contribution is negligible. To further reduce the noise, other methods mentioned above can be applied.

## 3. Nanomaterials used in PA stimulation

Our group has developed various high-efficiency PA emitters taking advantage of different nanomaterials and demonstrated their features in various neural stimulation applications. As is shown in figure 1, here we summarize 4 types of nanomaterials used in the corresponding PA devices with different characteristics.

To achieve omnidirectional PA emission for efficient neuromodulation both *in vitro* and *in vivo*, we developed a fiber optoacoustic converter (FOC) featuring a two-layer nanocomposite structure (figure 1(b)) [27]. The first layer, composed of ZnO nanoparticles (15% w/w in epoxy) with a diameter of 100 nm, serves as a diffusion medium to scatter the incident laser light and facilitate omnidirectional emission. A 200 *µ*m optical fiber was dipped 150 *μ*m into the mixture and quickly pulled out, and then cured at room temperature for 30 mins to get coated. For efficient PA generation, a second layer containing graphite powder (30% w/w in epoxy) was coated by the same method to absorb light, leveraging graphite’s high optical absorption coefficient and thermal conductivity. With this two-layer design, the FOC achieved a peak acoustic pressure of 0.48 MPa at a pulse energy of 14.5 *μ*J, corresponding to a conversion efficiency of 1.04 (kPa $ \cdot $ mm^2^) *μ*J^−1^.

To improve conversion efficiency and spatial resolution enabling multi-site stimulation and single-cell modulation in mechanistic studies, we further studied other nanomaterials. CS, a carbon-based material typically composed of 50–100 nm particles and formed during incomplete combustion under low-oxygen conditions, was chosen for its superior optical absorption and its ability to diffuse into the thermal expansion materials, such as polydimethylsiloxane (PDMS) [28]. Owing to its dense deposition, the absorption of the deposited CS layer is 5–10 times higher than that of graphite. Additionally, the epoxy matrix was replaced with PDMS, which has a 4–10 times larger thermal expansion coefficient to further enhance PA conversion efficiency. A 200 *μ*m fiber was inserted into a fiber ferrule and placed into the flame core of a paraffin wax candle to get fully coated with flame synthesized CS. Then, a nanoinjector was used to deposit a controlled amount of PDMS (∼0.01 *μ*m^3^) onto the tip of the fiber coated with CS. With these material improvements, we developed a CSFOE, capable of generating a PA pressure of 9 MPa at a pulse energy of 56 *μ*J (figure 1(b)). This corresponds to a conversion efficiency of 5.46 (kPa $ \cdot $ mm^2^) *μ*J^−1^—approximately five times greater than that of the FOC.

With the improved PA conversion efficiency, we developed a next generation of fiber-based PA emitter with enhanced spatial resolution for single-cell-level stimulation [29]. The optical fiber was tapered to a diameter of 20 *μ*m to match the typical size of a single neuron. However, this small diameter posed challenges for direct coating with CS through flame synthesis. To address this, and to balance the optical absorption and viscosity required for effective coating, we formulated a CNT and PDMS composite (figure 1(b)). For PDMS, the silicone elastomer was dispensed directly into a container carefully to minimize air entrapment, followed by mixing with the curing agent in a ratio of 10:1 by weight. The CNT concentration was increased to 15% (w/w) in PDMS to improve both light absorption and the viscosity of the mixture, so it could be applied to the tapered fiber tip using a punch-through coating method. The coating mixture was cast on a metal mesh to form a uniform film, which is punched through by the fiber controlled by a 3D micromanipulator to get a layer transferred to the tapered end. Using this approach, the resulting tapered fiber optoacoustic emitter (TFOE) achieved a final tip diameter of 19.8 *µ*m and generated a peak PA pressure of 2.7 MPa at a pulse energy of 6.7 *μ*J, corresponding to a conversion efficiency of 0.13 (kPa $ \cdot $mm^2^) *μ*J^−1^.

Toward minimal invasiveness and improved specificity, a semiconducting polymer nanoparticle-based PA nanotransducer (PAN) was developed for neural stimulation both *in vitro* and *in vivo* (figure 1(b)) [30]. Near infrared (NIR-II) absorbing semiconducting polymer bis-isoindigo-based polymer (BTII) is first synthesized. Then, it was modified with polystyrene-block-poly(acryl acid) (PS-b-PAA) via a nanoprecipitation method to obtain nanoparticles. The size of the prepared nanotransducers was measured to be 52.9 **±** 12.2 nm by transmission electron microscopy imaging. The PANs were found to bind onto the neurons at an estimated density of 40.2 **±**15.9 PANs per soma at a culture concentration of 2 *μ*g ml^−1^ . Besides, PAN can also be biconjugated with antibodies to specifically target the mechanosensitive ion channel transient receptor potential cation channel subfamily V member 4 (TRPV4). Through this method, PAN realizes ion channel–specific neuromodulation. Under a 1030 nm nanosecond laser with a pulse width of 3 ns, a repetition rate of 3.3 kHz, and an energy density of 21 mJ cm^−2^, a 1.0 mg ml^−1^ nanoparticle solution generates a PA signal with a width of 2 *μ*s and a peak-to-peak amplitude of 33.95 mV. The peak pressure generated by PAN at the concentration of 1 mg ml^−1^ was measured to be 1.36 kPa under the laser power density of 21 mJ cm^−2^ using a needle hydrophone, which has a noise limit of 143 Pa.

In summary, different nanomaterials have been investigated and used for neuromodulation, enabling improvements in PA conversion efficiency, spatial resolution, and cell specificity. While investigating different nanomaterials in the various emitters lays out a path to optimize for higher PA efficiency, functions such as biocompatibility, flexibility, multi-function, and non-invasiveness are also significant features that need to be considered for meaningful biomedical applications [31]. Moving forward, we aim to further explore the promising combination between nanomaterials and PA technologies to expand their capabilities in neuroscience and beyond.

## 4. **Development of PA interfaces and their applications** ***in vitro***

### 4.1. Fiber-based PA stimulation devices with high spatial resolution

A variety of interface designs have been developed to generate PA signals, such as fiber, film, and nanotransducer. Among these, fiber-based emitters are particularly promising for high-resolution neuromodulation applications due to their inherently small diameters, which enable stimulation at the single-neuron and subcellular level. Our group has demonstrated the development of fiber-based optoacoustic emitters [27–29]. FOE is made of a commercially available optical fiber with typical diameters in the range of 20–200 *μ*m. The distal end of the optical fiber was coated with PA materials, including nanocomposites, i.e., mixtures and layers of nanomaterial and elastic materials. We discuss the specific device designs and their applications as follows.

**Figure 2.**
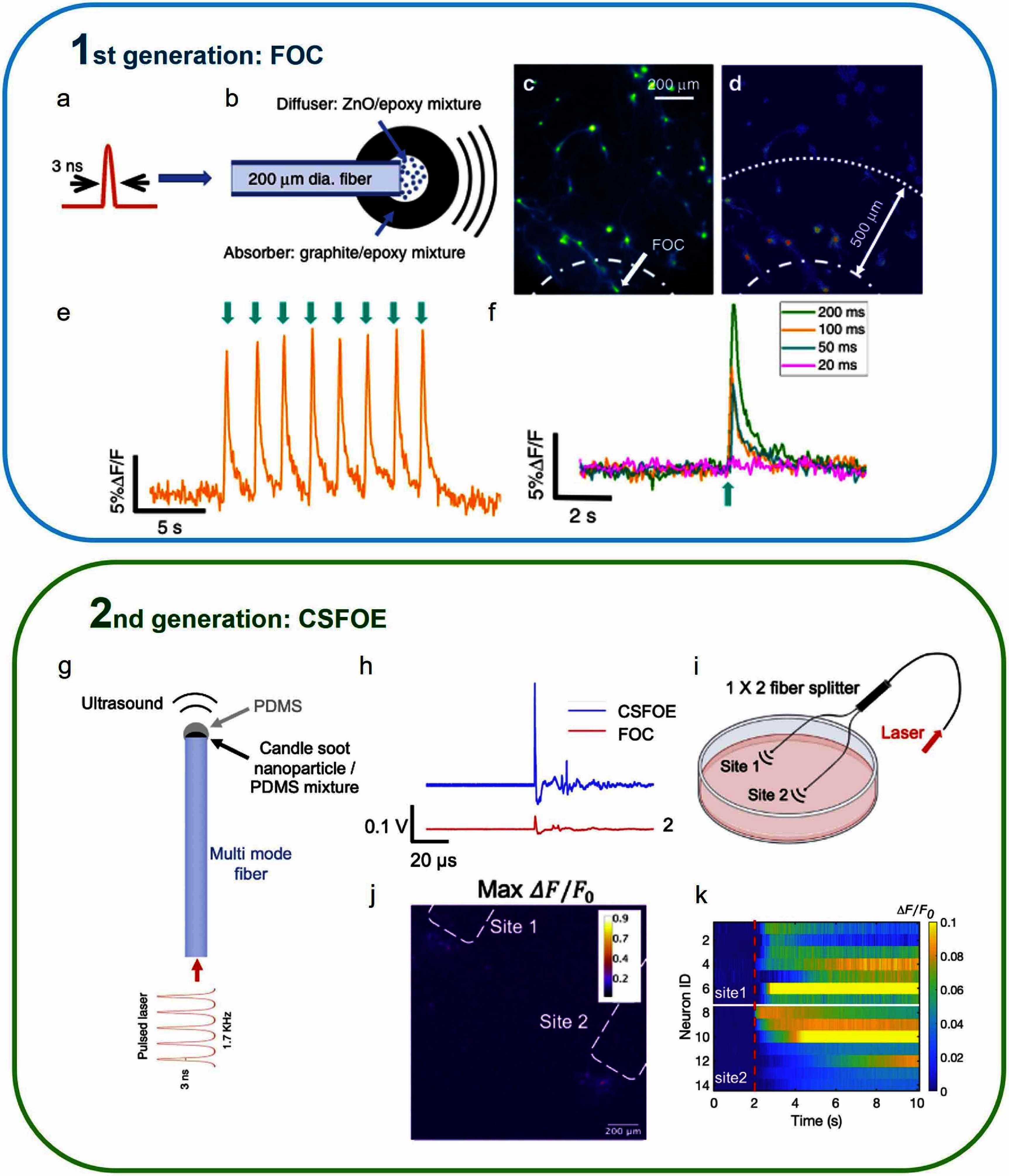
Two generations of fiber-based PA emitter *in vitro* validation. (a) and (b) Schematic of an acoustic wave generated by FOC from a nanosecond laser. (c),(d) Spatial distribution of calcium response and maximum neuronal calcium change induced by 200 ms FOC stimulation. Dashed line: placement of FOC. Dotted dashed line: 500 *μ*m away from the FOC. (e) Calcium trace of a neuron undergone repeated FOC stimulations. Green arrows: laser burst onset. (f) Representative Ca traces of neuronal response to 200 ms, 100 ms, 50 ms, and 20 ms FOC stimulation. (g) Schematic of acoustic wave generation by CSFOE. (h) Photoacoustic signal of CSFOE and FOC, measured by a 5 MHz transducer under the same laser condition: 1030 nm, 3 ns, 1.7 kHz, 48 mW. (i) Schematic of dual-site stimulation using two CSFOEs with a fiber splitter. Created with BioRender.com (j) Max Δ*F*/*F*_0_ image of neurons at two sites stimulated by two CSFOEs. (k) Colormaps of fluorescence changes in 14 neurons at two sites stimulated by CSFOE. Reproduced from [27], with permission from Springer Nature. Reproduced from [28]. CC BY 4.0.

The first generation of fiber-based optoacoustic emitters (FOE, also referred to as fiber-based optoacoustic converter, FOC) was made of a 200 *μ*m optical fiber (200EMT, Thorlabs), with a coating at the tip resulting in a total diameter of 600 *μ*m [27]. The pulsed laser was a passively Q-switched diode-pumped solid-state laser (PQSY, RPMC USA) centered at 1030 nm with a pulse width of 3 ns, a repetition rate of 3.6 kHz and pulse energy of 100 *μ*J (figure 2(a)). As discussed in the session 3, the coating had two layers: a diffusion layer of a ZnO/epoxy mixture, and an absorption layer of a graphite and epoxy mixture (figure 2(b)). To confirm whether the FOC can directly stimulate neurons, rat cortical neurons (days *in vitro* 18–22) with a calcium indicator, Oregon Green™ 488 BAPTA-1 dextran (OGD-1) were studied. Calcium imaging was performed on the neuron culture to record the neuron activities. When the FOC was applied to the neuron culture with a laser pulse train of 200 ms duration and repetition rate of 3.6 kHz, the generated localized ultrasound elicited a detectable Ca^2+^ response in neurons (figures 2(c) and (d)). The neuron within 500 *μ*m showed a strong calcium response with the maximum Δ*F/F*_0_ of 9.4 ± 3.4%, while the neuron at 500 *μ*m to 1 mm showed a weaker response with the maximum Δ*F/F*_0_ of 1.5 ± 1.0%. This result indicates that the PA stimulation through FOC is highly localized with a spatial resolution of 500 *μ*m *in vitro*, comparable to the diameter of FOC. To confirm whether the FOC can stimulate the neuron repeatedly, 8 laser pulse trains with an interval of 2 s were delivered to the FOC. Each laser pulse train caused a repeatable calcium response (max Δ*F/F*_0_ = 12 ± 0.6%, figure 2(e)). Next, the laser pulse duration was changed to 100 ms, 50 ms, and 20 ms to find the threshold for FOC-induced neural activation (figure 2(f)). Under the specific laser condition, FOC with a laser pulse duration of 100 ms and 50 ms can successfully activate neurons, while FOC with the pulse duration of 20 ms cannot evoke responses.

CSFOEs were then developed as the second generation of fiber-based PA emitters with the goal to improve PA conversion efficiency [28]. Through simulation and experiments, we have demonstrated a layered PA coating, i.e., a CS and PDMS mixture layer and a PDMS layer to offer more efficient conversion (figure 2(g)). Under the same pulse laser power of 48 mW, the CSFOE generates up to a 9.6 times stronger PA signal than the FOC (figure 2(h)). This result showed that CSFOE, benefiting from better material properties offered by CS and PDMS and a layered design, is a more efficient PA emitter, enabling safer neural stimulation and new functions. Specifically, CSFOE has been used as a dual-site neuron stimulator driven by a single pulse laser (figure 2(i)). The laser energy is evenly divided to 53 *μ*J, corresponding to a pressure of 8.5 MPa, through a 1 × 2 fiber splitter for each target site. Laser repetition rate was 1.7 kHz, and duration was 3 ms. Monitored by Ca imaging, GCaMP6f-labeled primary neurons (days *in vitro* 10–13) at both sites within a distance of ∼200 *μ*m were successfully activated by CSFOE. A fluorescence increase of around 10% was observed (figure 2(j)). Representative traces of 14 cells from two sites are plotted in the map of fluorescence changes, and successful stimulation can be clearly seen with the increase in fluorescence intensity *ΔF/F*_0_ > 10% (figure 2(k)) after the laser on (the red dashed line).

**Figure 3.**
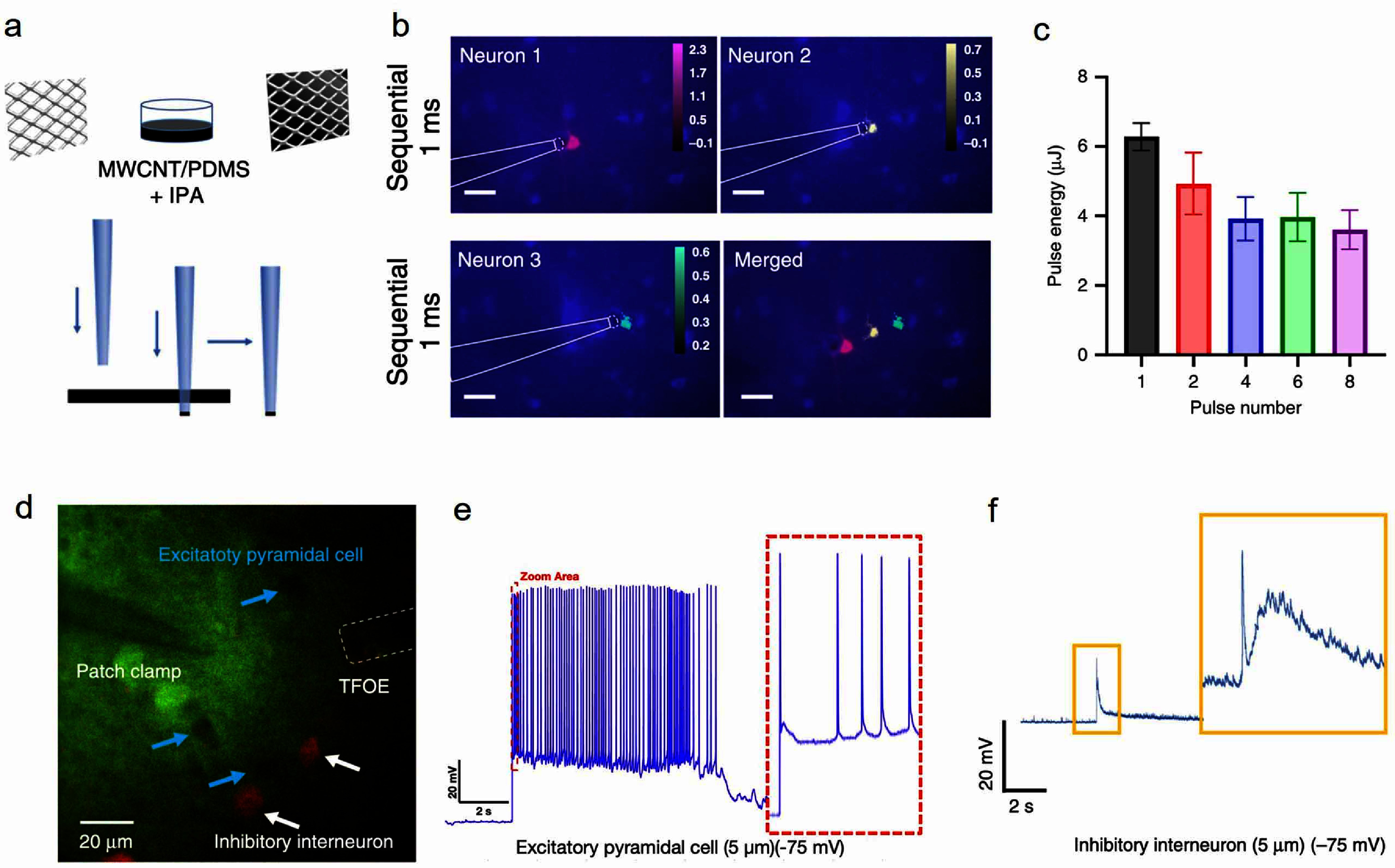
Tapered fiber-based PA emitter stimulates neurons with single neuron resolution and evokes e-phys response on a brain slice. (a) Schematic of TFOE fabrication. (b) Sequential stimulation of three neurons. Max Δ*F*/*F*_0_ labeled in red, yellow, and green, respectively, superimposed with the fluorescence image. Scale bars: 50 *μ*m. (c) Pulse energy threshold for successful neuron stimulation as a function of pulse number (*N* = 5–7). (d) Two-photon imaging of patch clamp integrated with TFOE in a mouse brain slice targeting GAD2-tdTomato negative pyramidal neurons and GAD2-tdTomato positive inhibitory interneurons. The patch pipette is visualized using the cyan-green fluorescent dye Alexa Fluor 488 in the intracellular electrode solution. (e),(f) TFOE single neuron stimulation integrated with whole cell patch clamp. Membrane voltage responses in (e) excitatory pyramidal cell and (f) inhibitory interneuron upon TFOE stimulation at a distance of 5 *μ*m are presented. Reproduced from [29], with permission from Springer Nature.

PA stimulation with single neuron resolution is an important feature as it offers a new tool in fundamental studies, understanding how neurons respond to mechanical stimulation, and offers the capability required for specific clinical applications. To this end, we have developed a TFOE that generates a localized ultrasound field of 39.6 *μ*m. Specifically, a tapered fiber with a tip diameter of ∼20 *μ*m was first obtained by pulling a commercial optical fiber with a diameter of 225 *μ*m [29]. A CNT/PDMS mixture was coated on the tapered fiber tip through a lab developed punch-through method (figures 1 and 3(a)).

To illustrate the spatial resolution of the TFOE, primary rat cortical neurons expressing GCaMP6f were cultured, and calcium imaging was performed using an inverted wide-field fluorescence microscope. The delivered laser was 3 ns, 7.8 mW at 1030 nm with a repetition rate of 1.7 kHz. Here, three neurons with an edge-to-edge spacing of 25 ± 2 *μ*m were selectively targeted. The TFOE was placed ∼5 *μ*m away from each targeted neuron. The laser duration is 1 ms, and the interval between each recording is 1 min. The maximum fluorescence intensity change was color-labeled for each neuron in pink, yellow, and green, respectively (figure 3(b)). It can be seen obviously that only the targeted neuron was stimulated, without any simultaneous effect on the other two neurons, which means that TFOE can provide single-neuron spatial resolution in neuromodulation. Apart from this, the TFOE shows high temporal resolution as well. When the laser pulse energy reaches 6 *μ*J, a generated single ultrasound pulse with a width of less than 1 *μ*s can activate the neuron successfully, which shows an unprecedented temporal precision compared to ultrasound neural stimulation using transducers (figure 3(c)). If more than one pulses were used, the threshold of stimulation decreased and reached a plateau of 3.9 *μ*J when the pulse number was greater than 4 pulses.

Furthermore, neuron response to TFOE stimulation can be recorded using the intracellular patch-clamp owing to its high spatial resolution and minimized mechanical disruption. Mouse cortex slices expressing tdTomato in GAD2 interneurons were used. Specific cell types: GAD2-tdTomato positive inhibitory interneurons and GAD2-tdTomato negative pyramidal neurons were visualized (figure 3(d)). TFOE was integrated with the patch pipette to induce depolarization, which leads to action potential generation in the targeted neurons. A series of action potentials occurred on the excitatory pyramidal cells right after the TFOE stimulation (figure 3(e)), while inhibitory interneurons showed a sharp peak and a following broad peak at the same stimulation condition (figure 3(f)). This result suggested the two cell types have different intrinsic action potential thresholds due to different distributions of mechanosensitive ion channels, which leads to have different response dynamics to acoustic radiation force.

### 4.2. Film-based PA stimulation devices with single-neuron resolution and biocompatibility

Biocompatible film is an emerging platform for brain-machine interfaces to modulate and record brain response [32–34]. Through integrating photon absorbers into a film, a PA film can be fabricated to generate ultrasound and modulate neurons via the PA effect [35, 36]. Compared to fiber-based PA emitters, films can cover a larger area and therefore are suitable for high-throughput neural modulation and screening. Besides, the recent advances in film-tissue interface endow versatile functionalities of the film, including tunable mechanical and optical properties, controlled degradability, and bio-adhesiveness [37–41]. Together with these unique properties, a multifunctional film-based PA neural interface can be designed to serve different biomedical applications, including neural signal recording, structural support, neural modulation, and regeneration.

**Figure 4.**
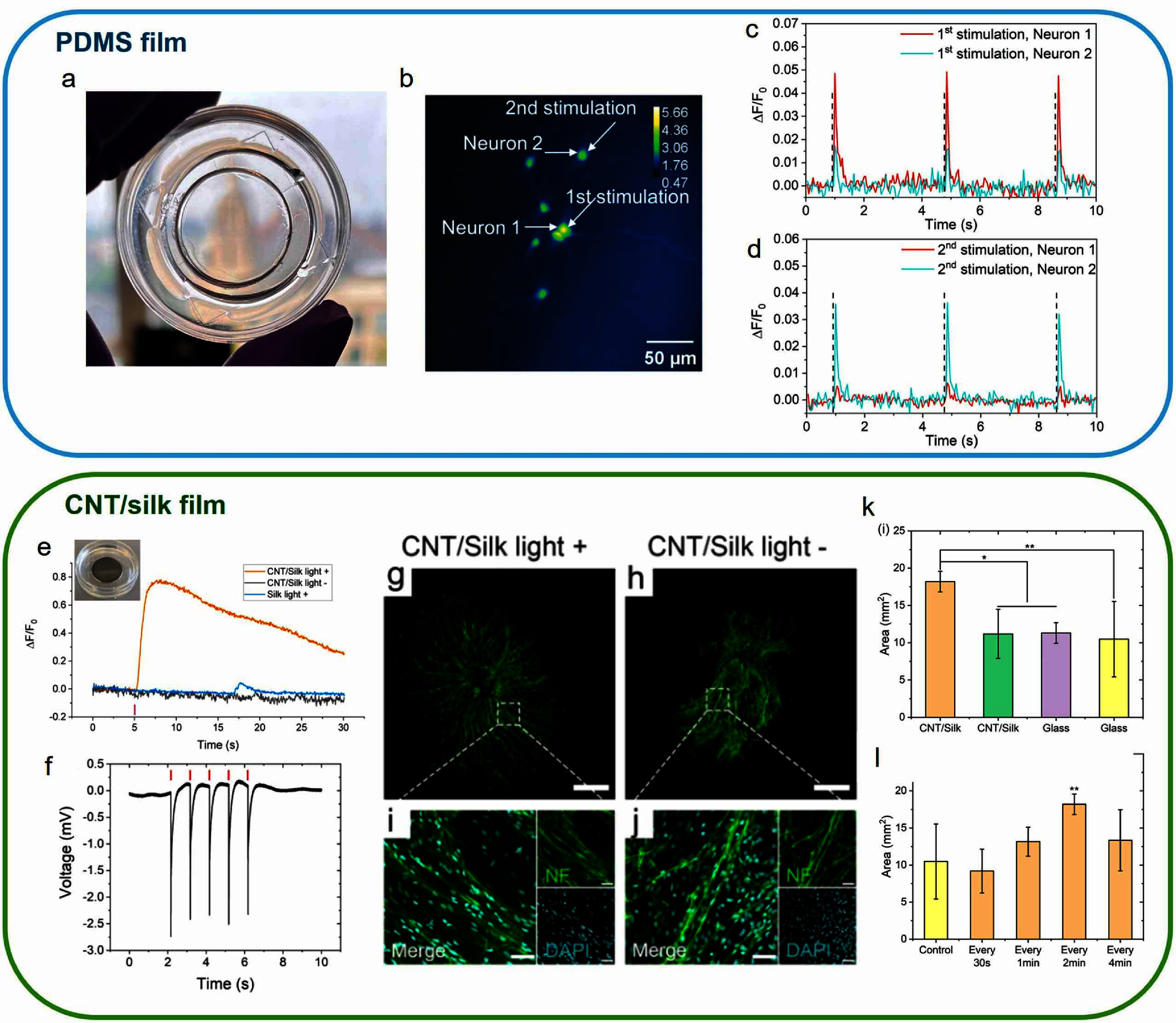
Film-based PA emitter *in vitro* validation using PDMS and silk-CNT. (a) Photograph of PDMS PA films. (b) Representative calcium image of Oregon green labeled cortical neurons at DIV12 cultured on the PDMS film before sequential MIPA stimulation. White arrows indicate the targeted neurons. The color scale represents relative fluorescence intensity. (c)–(d) Corresponding calcium traces of Neuron1 and Neuron2 during two sequential MIPA stimulations in b. Black dashed lines: the laser onset. (e) Photograph of CNT/silk PA film (inset) and representative calcium traces of neurons cultured on the CNT/silk film within the laser illumination area (light +) and out of the laser illumination area (light −) and cultured on a silk film within the laser illumination area (silk light +). (f) Extracellular recording of DRG explants cultured on the CNT/silk film. Red lines: five repeat photoacoustic stimulations were performed with a laser pulse energy of 14.7 *μ*J. (g),(h) Representative confocal images of DRGs stained for neurofilament (green): DRGs cultured on CNT/silk film (g) with laser illumination and (h) without laser illumination. Scale bar: 1 mm. (i), (j) High-resolution confocal images of DRGs stained with Anti-Neurofilament 200 (NF) for neurites (green) and DAPI for nuclei (cyan). Scale bar: 50 *μ*m. (k) Average neurite coverage area for DRGs in four groups. (l) Average neurite coverage area for PA-stimulated DRGs with various stimulation frequencies. Control: DRGs cultured on glass without light stimulation (glass light −). Laser train duration of 5 ms, a 1.7 kHz repetition rate, and a pulse energy of 14.7 *μ*J. All DRGs were allowed to grow for 2 d before stimulation and were fixed at day 10. Error bars represent standard deviation (*n* = 5, **p < 0.01, *p < 0.05, one-way ANOVA and Tukey’s mean comparison test). [35] John Wiley & Sons. © 2024 The Author(s). Advanced Science published by Wiley-VCH GmbH. Reprinted with permission from [36]. Copyright (2022) American Chemical Society.

Traditional PA emitters rely on materials that have strong absorption in the visible and NIR range, such as metal, carbon materials, and perovskites, to absorb the energy from the incident laser [42–47]. Despite high PA conversion due to the strong absorption, the reduced transparency in the visible and NIR range caused by these absorbers makes it difficult to collect high quality optical signals from the same window. To solve this problem, our group recently reported a mid-infrared PA film (MIPA) via vibrational excitation in PDMS, a biocompatible material [35]. PDMS film is transparent in the visible range (figure 4(a)), which is compatible with high fidelity fluorescence image. In addition, the abundant C–H groups in PDMS efficiently absorb photons at 3.38 *μ*m wavelength with 7.8 *μ*m absorption depth through vibrational excitation. Such high absorption results in a PA conversion efficiency of 61.25 mV *μ*J^−1^, 37.5 times higher compared to traditional opaque carbon material-based PA film, indicating its potential to stimulate neurons with lower laser energy.

High precision PA neural stimulation was achieved with a focused mid-infrared laser (figures 4(b)–(d)). 50 *μ*m spatial resolution was proven through a sequential neural stimulation in the same field of view. As shown in figures 4(c) and (d), only targeted neurons showed stimulation. Notably, the MIPA stimulation is repeatable on the same neurons over 10 times without observable calcium signal loss and damage, indicating the reliability of MIPA stimulation. Together with high transparency, PA conversion, biocompatibility, and reliable PA neural stimulation with high precision, MIPA opens up opportunities for high-throughput neural stimulation.

In addition, our group presented the development of PA scaffolds combining biocompatible silk fibroin with CNTs [36]. The study demonstrates how a biocompatible film-based PA neural interface can promote both neuronal activation and tissue regeneration via PA stimulation.

The CNT/Silk film (figure 4(e)) developed in this study is a biocompatible porous scaffold composed of silk fibroin integrated with CNTs. The scaffold is designed to serve as both a structural support for regenerating neural tissue and a transducer that converts pulsed NIR light into localized acoustic waves through the PA effect. The silk fibroin serves as a biodegradable, biocompatible matrix that provides structural support for neural tissue, promotes cell adhesion, and facilitates mass transport. The CNT-embedded silk scaffolds efficiently converted pulsed NIR laser light into acoustic waves. These waves could be utilized to stimulate neurons without causing thermal damage, as confirmed by a thermal camera monitoring during the simulation. PA stimulation elicited responses repeatably and reliably in rat cortical neurons, confirmed by Calcium imaging (figure 4(e)) and by the extracellular electrical recording on the DRG explants (figure 4(f)).

Regenerative effect from PA neural stimulation was also investigated in the study. DRG explants subjected to PA stimulation showed significantly enhanced neurite outgrowth (figures 4(g)–(j)). After a single 1 h treatment (5 ms pulses every 2 min), stimulated DRGs exhibited a 1.74-fold increase in neurite coverage area compared to controls (figure 4(k)). The optimized stimulation frequency was found to be once every 2 min; more frequent stimulation (e.g. every 30 s) was less effective, possibly due to calcium desensitization (figure 4(l)). The promoted neurite outgrowth was due to the increased neurotrophic factor expression. It was found that PA-stimulated DRGs had nearly 2-fold increased levels of brain-derived neurotrophic factor (BDNF), a critical molecule in neural growth and synaptic plasticity. This activity-dependent BDNF expression aligns with known calcium-dependent signaling pathways triggered by PA stimulation. This work provides a powerful proof of concept that film-based PA scaffolds can serve dual roles in neural modulation and regeneration.

### 4.3. Minimal invasive polymer nanoparticle-based PA stimulation with single-cell resolution and specific targeting

Nanoparticle has been used as a vehicle in optical, magnetic, and acoustic induced neuromodulation [48–52]. PA nano-transducers (PANs) are based on NIR-II absorbing semiconducting polymer bis-isoindigo-based polymer (BTII). It can strongly absorb the nanosecond pulsed laser in the NIR second window (NIR-II; 1000–1700 nm) and generate localized acoustic waves [30].

**Figure 5.**
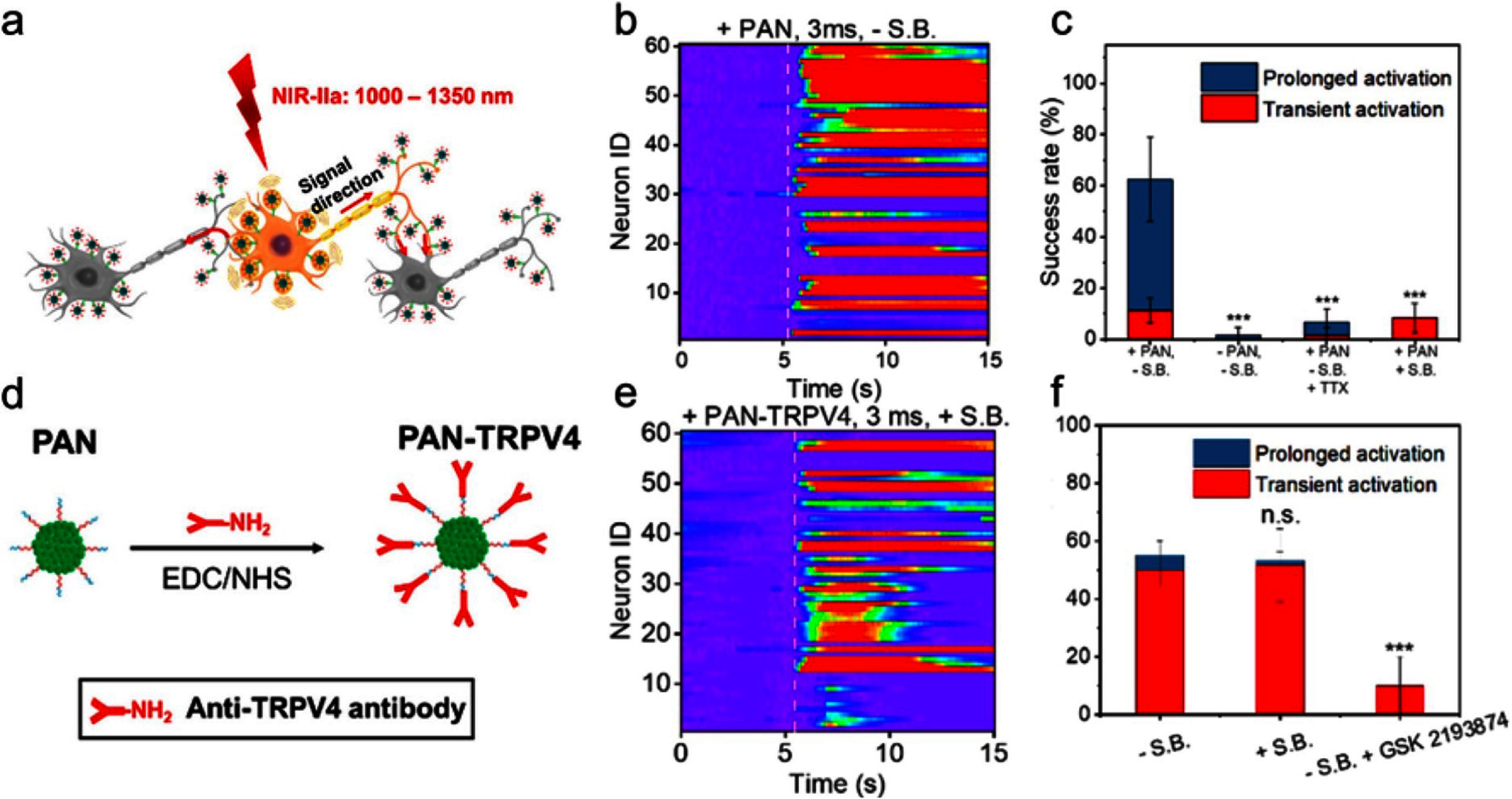
Nanotransducer stimulation *in vitro* with specific targeting. (a) Scheme of the PAN-induced neural stimulation. (b) Color maps of fluorescence changes in neurons stimulated by PANs with a 3 ms pulse train. White dashed lines indicate laser onsets. (c) Success rate analysis of PAN-induced neuron stimulation profiles in the presence/absence of synaptic blockers or TTX. Error bars denote the standard deviation (SD) (*n* = 60). (d) Schematic of PAN-TRPV4 synthesis. (e) Color maps of the fluorescence intensity change in neurons treated with PAN-TRPV4 without synaptic blockers. (f) Success rate analysis. Error bars denote SD (*n* = 60). Reproduced or adapted with permission from [30] (copyright 2021 Elsevier).

Two types of PAN were studied. Sprague-Dawley rat primary cortical neurons transfected with GCaMP6f were first incubated with 150 *μ*l of 20 *μ*g ml^−1^ PAN solution for 15 min, and confirmed with an estimated 40.2 ± 15.9 PANs bound per soma through charge-charge interaction. A nanosecond laser at 1030 nm with a pulse width of 3 ns, a repetition rate of 3.3 kHz, a duration of 3 ms and a laser pulse energy of 17 mJ per pulse (pulse energy density of 2.1 mJ cm^−2^) was delivered to the culture via a 200 *μ*m diameter optical fiber. 37 of 60 studied neurons showed a fluorescence increase of greater than 10% after the stimulation (figure 5(b)). Specifically, 11.2% were observed responding transiently, and 51.3% had prolonged responses, taking a longer time (up to 60 s) to recover to the baseline. After the addition of TTX, a blocker of voltage-gated sodium channels, only 6.7% of neurons showed activation upon laser excitation. A cocktail of synaptic blockers (10 *μ*m NBQX, 10 *μ*m gabazine, and 50 *μ*MDL-AP5) blocking the synaptic inputs decreases the transient activation success rate to 8.3% and completely blocks prolonged activation (figure 5(c)). These results indicated the transient activation is likely the result of direct PAN-mediated stimulation, while the prolonged activation comes from action potentials resulting from the activation of neural networks.

The second type of PAN studies was PAN bioconjugated with antibodies to specifically target the mechanosensitive ion channel TRPV4 (PAN-TRPV4). TRPV4 was chosen because of its high expression rate on the neuronal cell membranes and its capability of sensing external mechanical stimuli. Under the stimulation of PAN-TRPV4, 55% neural activations were induced (figure 5(e)). The success rate remained 53.3% with the application of the synaptic blocker cocktail. After adding the TRPV4 channel blocker, GSK 2193 874, only 10% of the neurons showed a transient response, and no prolonged activation was detected. These results indicated that PAN-TRPV4 induces more direct activation through specific targeting without significant involvement of the neural network and synaptic transmission.

## 5. Application of PA stimulation *in vivo*

Investigation of PA stimulation *in vivo* has been focused on activation of brain and retina, for their significance in potential clinical applications. Specifically, work has been performed to evaluate the spatial resolution of stimulation in brain and to validate successful stimulation in different brain regions and retina. The design goals of the interfaces were to develop strategies for implantation and non-invasive devices, and to integrate multiple functions on miniaturized interfaces.

### 5.1. Implanted fiber-based PA emitters directly evoke brain activities in rodent brain

**Figure 6.**
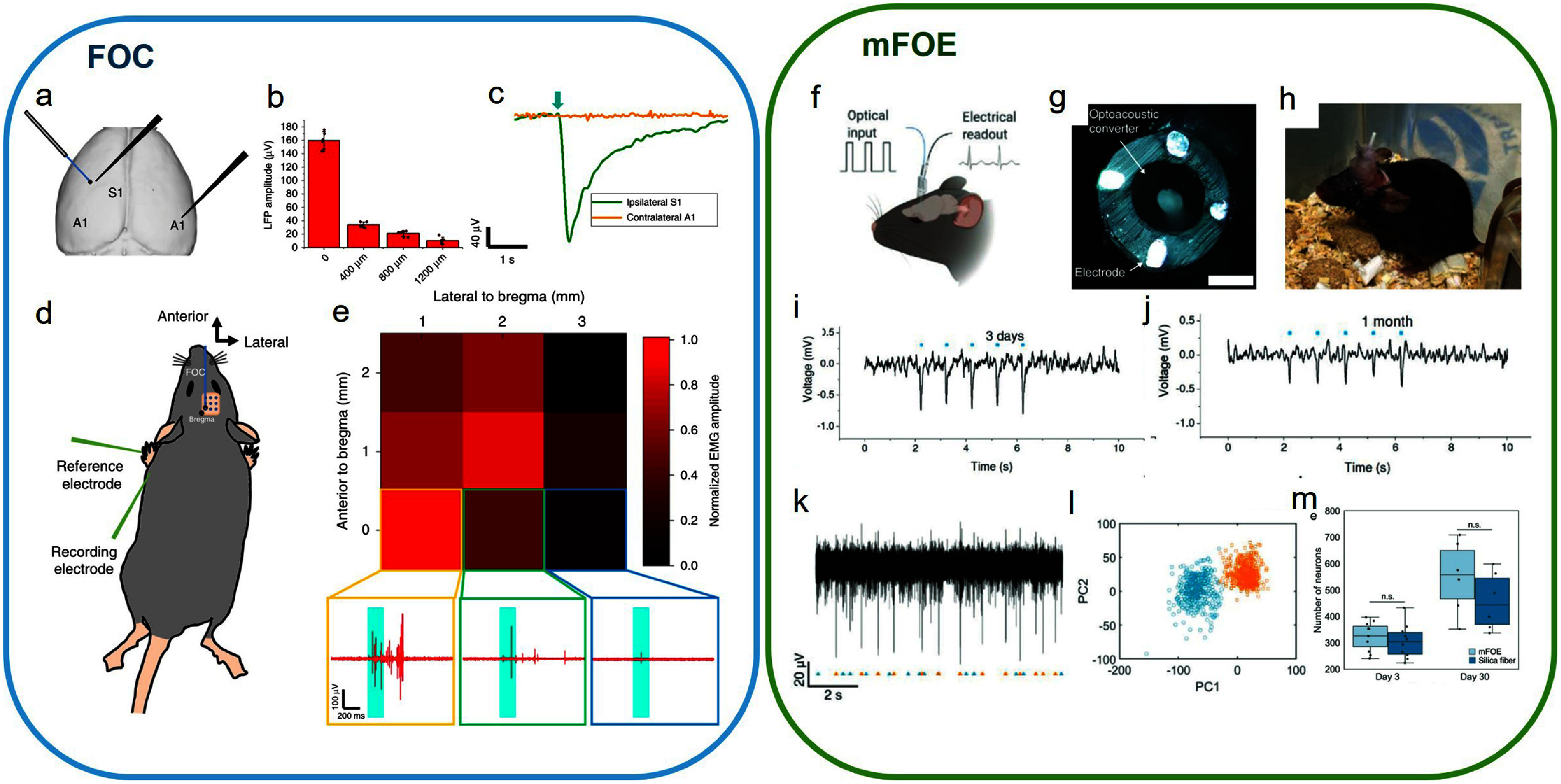
Fiber-based PA stimulation of rodent brains. (a) Placement of FOC in S1 and recording electrode in ipsilateral S1 and contralateral A1 to test the involvement of the auditory pathway. (b) LFP amplitude recorded at different distances from the FOC. Error bar,±SD.(c) LFP response pf ipsilateral S1 and contralateral A1 to S1 FOC stimulation. Green arrow: stimulation onset. d) Schematic of the motor cortex mapping experiment. (e) Heat map showing maximum peak-to-peak EMG response amplitude to FOC stimulation on different locations of the motor cortex. Inserts: representative EMG traces from indicated locations. (f) Illustration of the mFOE-enabled bidirectional neural communication. (g) Top view of the mFOE. Scale bar:100 *μ*m. (h) mFOE was implanted into the hippocampus of a C57BL/6 J mouse. (i), (j) Simultaneous optoacoustic stimulation and electrophysiological recording performed at (i) 3 d and (j) 1 month after implantation. Blue dots, the laser pulse trains. (k) Part of the filtered spontaneous activity containing two separable groups of spikes recorded by an mFOE electrode 1 month after implantation. (l) Principal components analysis of the two groups of spikes. m) Number of neurons in the field of view with implanted mFOEs, calculated by counting the NeuN-positive cells for mFOE and silica fiber at 3 d and 1 month after implantation (*n* = 10). Reproduced from [27], with permission from Springer Nature. [53] John Wiley & Sons. © 2023 The Authors. Advanced Healthcare Materials published by Wiley-VCH GmbH.

FOC evokes direct activation in the mouse brain [27]. Cranial windows were made above the primary somatosensory cortex (S1) and primary auditory cortex (A1) of mice based on stereotaxic coordinates with the dura intact. A laser pulse train of 200 ms duration was delivered to the FOC, and neural activities were recorded with a tungsten electrode. When targeting the S1 region, strong local field potential (LFP) responses of 159.8 ± 13.2 *μ*V were seen, demonstrating successful stimulation of the cortex. To quantify the spatial precision of stimulation, we recorded the LFP responses as the FOC moved away from the recording electrode. The LFP amplitude dropped to only 6.5% at 400 *μ*m away (*n* = 3) (figure 6(b)), demonstrating superior spatial confinement of FOC stimulation *in vivo*. Significantly, while the FOC evoked a robust LFP response on ipsilateral S1, no signal was recorded in the contralateral A1 (figure 6(c)). This indicated that the FOC induced direct neural stimulation *in vivo* with high spatial precision, without the involvement of the auditory pathway.

With sub-millimeter spatial precision of FOC’s brain stimulation, the response on the motor cortex was mapped by spatially scanning the FOC at different locations. A 3 × 3 mm^2^ stimulation area with 1 mm spacing covering the majority of the motor cortex was scanned by an FOC, and an electromyography (EMG) electrode was inserted subcutaneously and parallel to the triceps brachii muscle for recording (figure 6(d)). The maximum EMG responses were recorded at AP (anterior/posterior) 0, ML (medial/lateral) 1, and AP 1, ML 2, which is in agreement with previous motor cortex mapping studies. The results showed that the FOC can differentially modulate the mouse motor cortex at submillimeter spatial precision, which is a breakthrough compared with transcranial ultrasound neural modulation.

In addition to the cortex, FOE can modulate the deep brain region. A multifunctional fiber-based optoacoustic emitter (mFOE) has been developed as a bidirectional neural interface, enabling both stimulation and electrophysiological recording targeting the deep brain regions [53] (figure 6(f)). The polymer-based mFOE was integrated with three functional elements: (1) a center optical waveguide for light delivery, (2) an optoacoustic emitter layer, and (3) conductive recording electrodes (figure 6(g)). The waveguide was formed by polycarbonate core (PC, refractive index nPC = 1.586, diameter = 150 *μ*m) and polyvinylidene difluoride cladding (PVDF, refractive index nPVDF = 1.426, thickness = 50 *μ*m). Both polymers show a much lower Young’s modulus than the conventional silica fiber, mitigating the mismatched mechanical properties to the native neural tissue. The waveguide end was selectively coated with a PDMS layer mixed with carbon black, which acts as the PA transducer. Four electrodes were made of conductive BiSn alloy with diameters of 35 *μ*m, enabling localized electrophysiological recordings. This unique configuration allows the mFOE to transduce pulsed light into ultrasonic pressure waves for neural stimulation while simultaneously recording extracellular neural signals.

To evaluate the mFOE as a bidirectional neural interface capable of simultaneous neural stimulation and electrophysiological recording, the mFOE was chronically implanted into the hippocampus of wild-type C57BL/6 J mice (figure 6(h)). Laser pulses (1030 nm, 3 ns pulse width, 41.8 *µ*J energy, 1.7 kHz repetition rate) were applied in 50 ms bursts to generate localized PA waves. Electrophysiological recordings of LFPs and spikes were collected at multiple time points—3 d, 7 d, 2 weeks, and 1 month post-implantation. The mFOE successfully evoked LFP responses at all tested intervals (figures 6(i)–(j)). High-quality spike recordings were demonstrated using a principal component analysis and cluster isolation method, identifying distinct neuronal units (figures 6(k)–(l)). Immunohistochemical analysis compared mFOE to conventional silica fibers, showing reduced acute glial response and similar long-term biocompatibility.

These findings validated the mFOE’s chronic stability, biocompatibility, and ability to function as a bidirectional neural interface. Its orthogonal stimulation and recording capabilities offer a promising platform for closed-loop neuromodulation and brain-machine interfaces, particularly in contexts where viral transfection or electrical stimulation artifacts present limitations.

### 5.2. Minimal invasive and non-invasive PA stimulation in rodent brain

**Figure 7.**
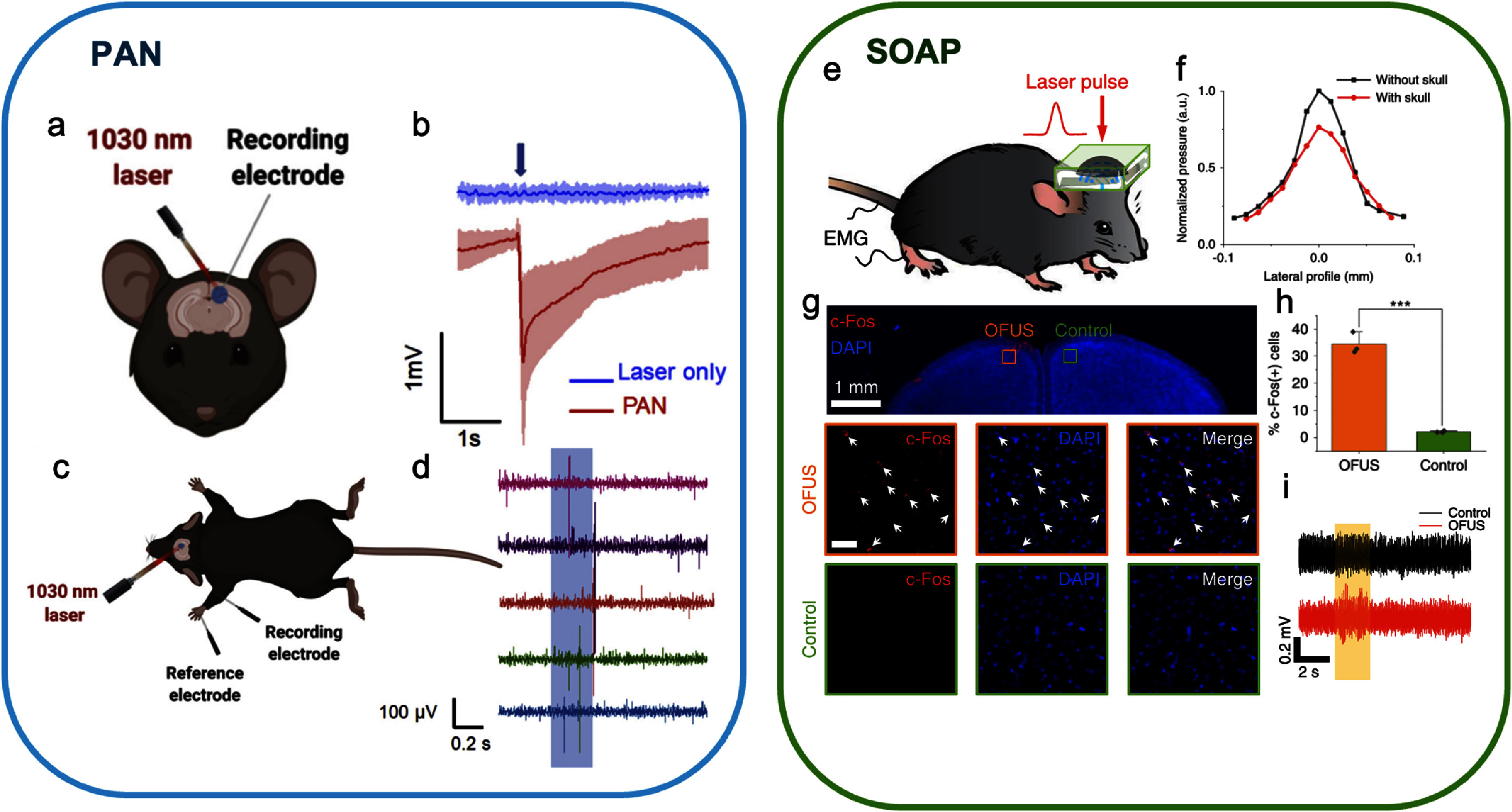
Minimum and non-invasive PA stimulation *in vivo*. (a) Schematic of *in vivo* electro physiology measurement with directly injected PAN. (b) Representative electrophysiology curves measured in the brain region without PANs as the control group (blue) and in the PAN-treated region (red) on three mice. The blue arrow indicates that the laser is on. (c) Schematic of EMG recording with directly injected PAN. (d) Forelimb EMG response to motor cortex PAN stimulation. (e) The schematic of OFUS stimulation *in vivo*. (f) The lateral resolution of OFUS without and with a piece of mouse skull. (g) Representative images of c-Fos and DAPI staining within the stimulation and control area. Red: c-Fos. Blue: DAPI. Orange outline: OFUS stimulated area. Green outline: control group at the contralateral area. Scale bar: middle and lower panel, 50 *μ*m. (h) Statistical analysis of the percentage of c-Fos positive neurons. ***p < 0.001, two-sample t-test. (i) Representative EMG recordings of 2 s OFUS stimulation and the control group targeting the somatosensory cortex. Orange box: laser on. Reprinted from [30], Copyright (2021), with permission from Elsevier. Reproduced from [26], with permission from Springer Nature.

Towards minimal invasive PA stimulation, PANs were tested for activating rodent brain [30]. The hair and skin on the dorsal surface targeted brain regions were trimmed. Craniotomies were made on primary motor cortex based on stereotaxic coordinates using a dental drill, and artificial cortical spinal fluid was administrated to immerse the brain. 600 nl of 1.0 mg ml^−1^ PAN solution was injected into the primary motor cortex of C57BL/6 mice. A 1030 nm, 3 ms laser pulse train with a pulse width of 3 ns, a repetition rate of 3.3 kHz and a pulse energy density of 21 mJ cm^−2^ produced a strong response in the stimulated cortex, while in the control group on the contralateral side, without PAN injection, the laser did not produce any response (figure 7(b)). PAN stimulation was effective in stimulating a behavior outcome, which was confirmed that by EMG measurement (figure 7(c)). At 1 h after the PAN injection, a needle electrode was inserted subcutaneously and parallel to the forelimb triceps brachii muscle. A reference electrode was inserted in the footpad with a ground electrode inserted subcutaneously on the trunk and ipsilateral to the stimulation site. A ground electrode was inserted subcutaneously on the trunk and ipsilateral to the stimulation site. A 200 ms laser pulse train was delivered to the injection site through an optical fiber. Strong EMG responses with an amplitude of 428.8 ± 119.0 mV and a delay of 127.8 ± 24.3 ms were recorded. The result suggests that PAN-mediated brain stimulation is sufficient to induce motor cortex activation and invoke subsequent motor responses.

Soft optoacoustic pad (SOAP) was developed as a non-invasive PA device enabling neuromodulation studies in rodent models [26]. Owing to the soft nature of the material, the PA film can be prepared as an ultrasound lens to focus the generated PA signal at a focal length of a few mm transcranially in the rodent brain. To achieve ultrahigh spatial resolution, the developed SOAP (figure 7(e)) had a numerical aperture of 0.95, approaching the theoretical limit. It achieved a lateral resolution of 66 *µ*m at the focal point and 83 *µ*m transcranially after penetrating a mouse skull (figure 7(f)), approximately half of the resolution achieved by a focused ultrasound transducer producing the same frequency.

SOAP’s neuromodulation capability *in vivo* was validated with immunofluorescence imaging and EMG. c-Fos immunolabeling revealed neuronal activation: adult C57BL/6 J mice treated with SOAP for 30 min at 33% duty cycle showed a localized c-Fos–positive region of ∼200 *µ*m in diameter (figure 7(g)). The proportion of c-Fos–positive cells was significantly higher on the stimulated side (34 ± 4%) compared to the control (2 ± 0.3%) (figure 7(h)). Functional outcomes were further confirmed by EMG, where stimulation of the motor cortex elicited strong hindlimb responses, unlike control stimulation of the somatosensory cortex (figure 7(i)). Together, these findings establish SOAP as the first non-invasive PA neuromodulation device capable of sub-0.1 mm spatial resolution, demonstrating both effective brain modulation and strong potential for neuroscience research and clinical translation.

### 5.3. Flexible PA implants evoke vision responses in rodent models: a case study for potential clinical application of PA stimulation

Retinal degeneration affects more than 200 million patients worldwide, yet no curative treatment currently exists. Retinal prostheses have therefore been developed to partially restore vision. For example, the Argus II system [54], based on electrical stimulation, provides only 60 pixels; the PRIMA photovoltaic device improves vision up to 20/500 acuity [55]; and POLYRETINA [56], another photovoltaic system, covers a visual angle of 43°. However, none of these approaches simultaneously achieves high spatial resolution and wide visual coverage.

**Figure 8.**
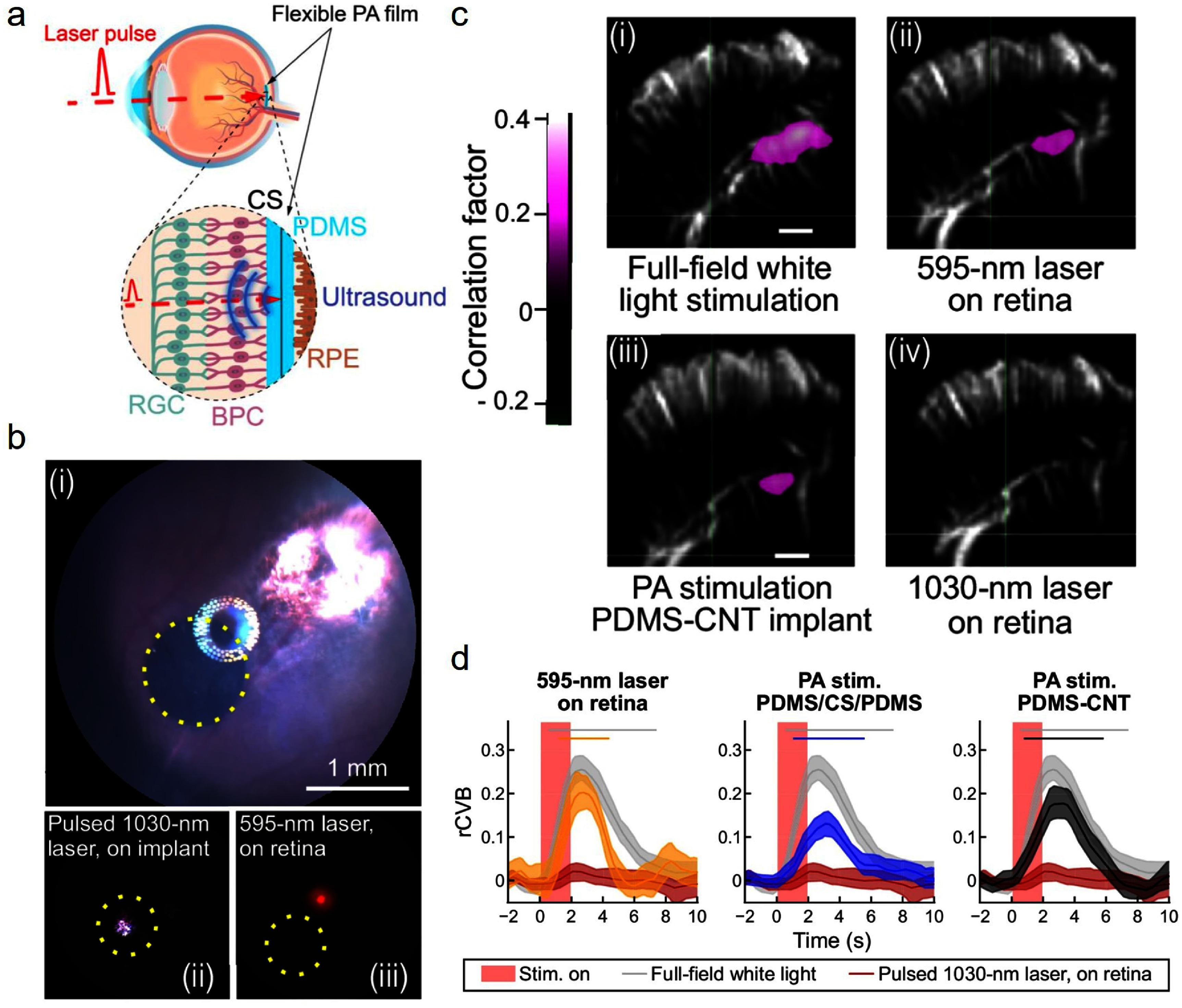
Retinal PA stimulation *in vivo*. (a) Working principle of the flexible photoacoustic (PA) film. Illumination of the PA film (cyan) with a nanosecond pulsed laser (red dashed line) produces an ultrasound field (blue). CS: candle soot, PDMS: polydimethylsiloxane, RPE: retinal pigment epithelium, RGC: retinal ganglion cells, BPC: bipolar cells. (b) Eye fundus images of a 1 mm PA implant (*i*, yellow dotted circle) and 400 *μ*m-diameter laser spots (ii: pulsed 1030 nm laser on the implant, iii: continuous 595 nm laser on the retina) used for laser and photoacoustic stimulation. (c) Functional ultrasound imaging in the coronal plane (left hemisphere, AP, −6.5 mm from the bregma). The correlation map displays the relationship between relative cerebral blood volume (rCBV) and the stimuli. Active pixels reflect regions of activated neurons in the contralateral superior colliculus (cSC) for a single recording (15 stimulations). (d) Mean rCBV responses of individual rats following stimulation with a laser (595 nm and 1030 nm on retina) and photoacoustic stimulation, for all rats. Horizontal bars denote significant elevation with respect to the baseline (e.g., no overlap of CI with basal CI). No significant difference in rCVB following photoacoustic stimulation between both implant types was found (e.g., overlapping confidence intervals). Peak rCBV values: white light (light gray), 0.26 (at 2.65 s); 595 nm (yellow), 0.20 (at 2.69 s); PDMS-CNT (dark gray), 0.18 (at 3.24 s); PDMS/CS/PDMS (blue), 0.13 (at 3.18 s); 1030 nm laser on retina (red), 0.02 (at 2.19 s). Shaded areas: 95% bootstrapped CI. Red shaded area: PA stimulation. Reproduced from [57], with permission from Springer Nature.

Characteristics of PA interfaces, including high spatial resolution, material flexibility, and scalability to large pixel counts through patterned light modulation, are particularly advantageous in retinal stimulation. A flexible PA film was recently developed as a retinal prosthesis. The film was shown to produce a highly confined ultrasonic field of 56 *µ*m upon a 50 *µ*m light illumination, demonstrating a potential high spatial resolution superior to existing retina prosthesis technologies. An implant with a size of 5 mm was shown to be successfully implated into the pig eyeball, a preferred model system for human eyeball, indicating its potential to offer a wide visual coverage due to its flexibility [57]. The film is designed for subretinal implantation in the degenerated photoreceptor layer. Upon laser irradiation, the film converts optical pulses into ultrasound, which stimulates the remaining mechanosensitive retinal tissue (figure 8(a)).

For *in vivo* studies, a 1 mm diameter film was implanted subretinally in Long-Evans rats. The experimental group received a 1030 nm laser targeted at the film, while control groups included 595 nm visible light on the retina and 1030 nm illumination outside the film (figure 8(b)). Stimulation consisted of a pulse train of eight 125 ms bursts over 2 s, repeated every 15 s, for a total of 15 stimulations per recording. Neural responses were monitored via functional ultrasound imaging at the superior colliculus. Full field white light stimulation, 595 nm laser on retina, and 1030 nm stimulation of the implanted film elicited significant activation, whereas 1030 nm stimulation on the retina alone produced no significant response (figure 8(c)). The mean relative changes in cerebral blood volume evoked by the PA film were comparable in amplitude to those induced by visible-light stimulation (figure 8(d)). These findings demonstrate that a PA retinal implant can effectively activate downstream visual pathways with response amplitudes comparable to visible light, highlighting its potential as a next-generation retinal prosthesis combining high spatial resolution and wide visual field coverage.

## 6. Understanding the mechanism of PA neural stimulation through *in vitro* studies

**Figure 9.**
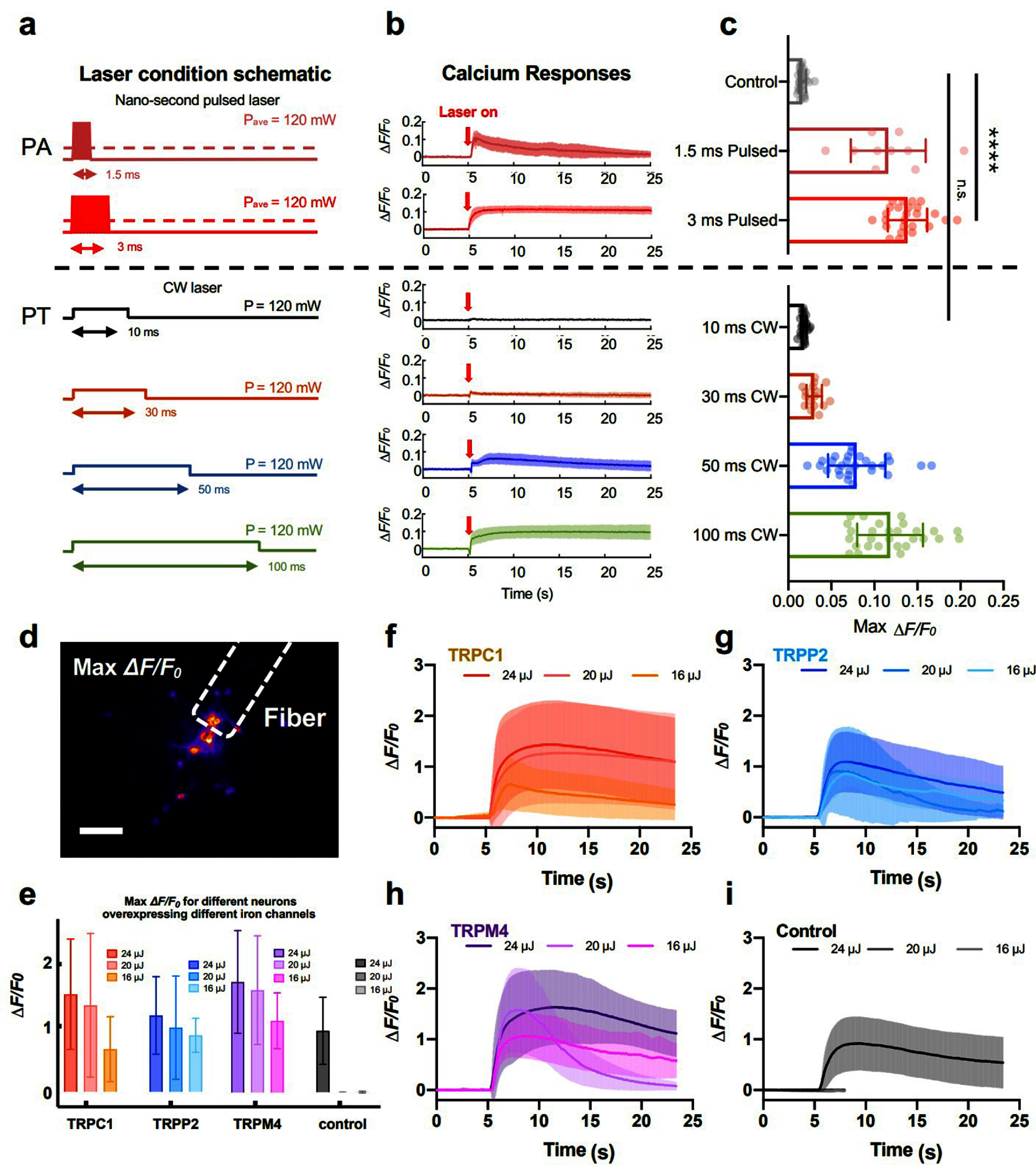
Mechanism of photoacoustic neuromodulation. (a) Schematic of different laser conditions used in PA/PT stimulation. Dark pink: pulsed laser with 5 pulses in 1.5 ms. Red: pulsed laser with 11 pulses in 3 ms. Black: CW laser with 10 ms duration. Orange: CW laser with 30 ms duration. Blue: CW laser with 50 ms duration. Green: CW laser with 100 ms duration. Average power of all laser conditions: 120 mW. (b) Ca^2+^ traces of neurons under different laser conditions shown in a. Laser on at *t* = 5 s (Red arrows). Solid lines: averaged traces. Shaded areas: SD. (c) Statistical analysis of maximum ΔF/F_0_ of neurons under PA and PT stimulation. *n* = 28, 10, 25, 28, 16, 28 for 1.5 ms pulsed, 3 ms pulsed, 10 ms CW, 30 ms CW, 50 ms CW, 100 ms CW, respectively. Control: no laser. *n* = 28 for control group, t-test, ****p < 0.0001. n.s. no significance. (d) Representative max Δ*F*/*F*_0_ contrast imaging of tapered FE-based PA stimulation with GCaMP6f labeled neurons. Dashed line: location of the tapered FE. Scale bar: 100 *μ*m. (e) Analysis of the calcium responses of neurons overexpressing ion channels upon FE stimulation with varied laser pulse energy. Error bars: SD. (f)–(i) Calcium traces of neurons overexpressing TRPC1 (*n* = 87)/TRPP2 (*n* = 28)/TRPM4 channels (*n* = 26) and control group (*n* = 55, recording was ended early due to no response observed) upon FE stimulation with varied laser pulse energy. Shaded areas: SD. Control: wild type. [13] John Wiley & Sons. © 2024 The Author(s). Advanced Science published by Wiley-VCH GmbH.

Understanding the mechanism underlying PA neuromodulation is of significant importance. Both PA and PT effects have been reported to modulate neuronal activities. During PA generation, both PA and PT fields are produced. To investigate the specific roles of these effects, we studied stimulation under two different laser stimulation conditions delivered to the same fiber-based PA emitter, i.e., a pulsed laser condition and a CW laser condition (figure 9(a)) [13]. The pulsed laser featured a 3 ns pulse width to ensure efficient PA generation in addition to the PT effect, while the CW laser produced primarily PT effects. In both cases, the average laser power was maintained at 120 mW, and the wavelengths were comparable (1030 nm for the pulsed laser and 1064 nm for the CW laser).

Given that the average power was constant between conditions, we assumed the resulting temperature increases to be similar. We applied both laser conditions to the same CSFOE while performing calcium imaging on cultured neurons labeled with OGD-488. As shown in figure 9(b), we observed distinct calcium responses depending on the laser condition. Under pulsed laser stimulation, neurons were efficiently activated with a total laser exposure of just 1.5 ms. In contrast, no response was observed with CW laser stimulation with an even longer laser duration of 10 ms. Increasing the pulsed laser duration to 3 ms resulted in overstimulation, characterized by prolonged calcium traces. To achieve a comparable effect using the CW laser, a duration of 100 ms was required—30–40 times longer than with the pulsed laser (figure 9(c)). These results indicate that the acoustic pressure generated in the PA process plays a dominant role in neuronal stimulation. The presence of PA stimulation lowered the energy threshold 30–40 times less compared to PT stimulation.

Although the temperature increase has been shown not to be the primary factor in PA neuromodulation, the mechanism of how acoustic waves modulate neuronal activity still remains unclear. Pharmacological studies were performed by blocking mechanosensitive ion channels in rat cortical neurons, which resulted in effectively suppressing responses. In addition, we overexpressed mechanosensitive ion channels TRPP2 and TRPC1, as well as the calcium-dependent amplifier TRPM4, and applied the same FOE-based stimulation protocol to the cultured neurons with GCaM6f labeling (figure 9(d)). As shown in figures 9(f)–(i), neurons overexpressing these mechanosensitive ion channels exhibited significantly higher Δ*F/F*_0_ responses compared to the control group. While the control group needs at least 24 *μ*J to stimulate neurons, the group with ion channel overexpression can lower the energy threshold down to 16 *μ*J. These results suggest that mechanosensitive ion channels are actively involved in the PA neuromodulation process.

## 7. Conclusion and outlook

Precise, long-term stable and safe modulation of neural circuits with high spatial and temporal resolution is needed for fundamental studies of brain functions as well as for clinical applications to address critical needs while drug treatments are not available. Here, nanomaterials and nanomaterial-based devices serve as the bridge connecting light absorption with acoustic transduction. They play a significant role in PA stimulation because of their strong absorption in the NIR window, high PA conversion efficiency, easy integration to various platforms, and biocompatibility.

In this paper, we summarize the progress made in using graphite, CNT, CS, and polymer nanotransducer to generate PA pressure efficiently. These nanomaterials were applied to different neural interfaces, such as implantable fiber emitters, flexible films, minimally invasive nanoparticles and non-invasive SOAP, for safe and effective neural stimulation. As is compared in table 1, the specifically designed TFOE, as a fiber-based interface, offers the best spatial resolution of 39.6 *μ*m and a fastest temporal resolution of 1 ms so far. SOAP, as an example of film-based methods, can achieve fully noninvasive (SOAP). Nanoparticle technology also shows a good performance with single-neuron resolution and a rapid 3 ms response. Each interface has its own strengths, and the optimal choice depends on the specific application requirements, such as the need for precise resolution, minimal invasiveness, or long-term stability [58].

**Table 1.** Comparison of performance for fiber-based, film-based, and nanoparticle-based PA stimulation.

	Fiber-based	Film-based [57]	Nanoparticle [30]
Spatial resolution	39.6 *µ*m (Single-cell) [29]	56 *µ*m	Single-neuron
Temporal resolution	1 ms [29]	51 ms	3 ms
*In vivo* region	Mouse brain [29]	Rat retina	Mouse brain
Stability	>1 month [53]	>90 d	n.a.
Noninvasiveness	Invasive [53]	Noninvasive	Minimally invasive

With successful PA modulation both *in vitro* and *in vivo*, we hypothesize that PA neural modulation is feasible in systems beyond the brain and retina. For example, peripheral nerves, muscles, and cardiovascular systems provide accessible and clinically relevant targets where PA-based neuromodulation could reveal new physiological principles and therapeutic opportunities. Understanding potential difference in PA sensitivity in these systems helps further clarify the mechanism of PA stimulation and extends the scope of neuromodulation to diverse organ systems for clinical applications.

Integration of nanomaterial-enabled PA stimulation with noninvasive readout technologies is important, particularly functional MRI. Combining stimulation and functional imaging on the accessible platform allows real-time mapping of brain responses, thereby establishing a closed-loop framework for both fundamental neuroscience and clinical translation. Furthermore, we would like to explore endogenous agents beyond synthetic nanoparticles, such as leveraging endogenous chromophores or bioengineered molecules, a new and attractive strategy to reduce toxicity and improve translational potential.

PA stimulation as initiated by light has an intrinsic feature providing the high spatial resolution, in principle, approaching diffraction limited spatial resolution of light. Single cell and sub-neuron resolution has already been demonstrated in multiple platforms [29, 30, 35]. However, its penetration depth is fundamentally limited also by light. Pulsed light with long wavelength at low tissue and water absorption, such as NIR, have been used to partially address this challenge. Fiber-based implantable interface with light coupled to the fiber directly can overcome penetration limitations at the expense of increased invasiveness. Importantly, we have designed SOAP, an interface that is essentially an optical driven ultrasound lens with the penetration depth defined by the device geometry and ultrasound wavelength generated, to enable a penetration depth beyond the light limitation. We will continue to explore strategies balancing precision, penetration and invasiveness. Despite that excitatory and inhibitory neurons in rat brain slices have shown distinct responses to PA stimulation, PA stimulation by nature is a non-genetic approach. At current stage targeting is achieved by spatial location and confinement of the generated PA field. Integration with sonogenetics can further enable its cell specificity as the cost of safety risk.

Success of non-genetic PA stimulation of rodent brain and retina has opened its potential in clinical application in treating neurologic disorders in brain or blindness. For example, PA retina prosthesis offers a potential resolution up to 56 micrometers and a scalability to integrate thousands of pixels over a footprint of a few mm^2^, exceeding the pixel density in the existing technologies. Such non-electrical neural interface can be more tolerant to scar formation and impedance changes, which could minimize degradation of the device performance. In addition, the flexible design opens its translational potential as a larger retina prosthesis enabling in large view angles necessarily supporting everyday activities of blind patents such as walking and navigation. In summary, for its technical merits of high precision, potential scaling-up ability, and excellent biocompatibility, nanomaterial based PA stimulation is expected to fuel up neural modulation studies to decode the complex neural circuits and lead to clinical translations.

## Data Availability

No new data were created or analyzed in this study.
